# Inhibition of NR2B-Containing N-methyl-D-Aspartate Receptors (NMDARs) in Experimental Autoimmune Encephalomyelitis, a Model of Multiple Sclerosis

**Published:** 2014

**Authors:** Mojtaba Farjam, Faegheh Baha'addini Beigi Zarandi, Shirin Farjadian, Bita Geramizadeh, Ali Reza Nikseresht, Mohammad Reza Panjehshahin

**Affiliations:** a *Department of Medical Pharmacology, School of Medicine, Fasa University of Medical Sciences, Fasa, Iran. *; b *Department of Pharmacology, School of Medicine, Shiraz University of Medical Sciences, Shiraz, Iran. *; c *Department of Immunology, School of Medicine, Shiraz University of Medical Sciences, Shiraz, Iran.*; d *Transplant Research Center, Department of Pathology, Shiraz University of Medical Sciences, Shiraz, Iran. *; e *Department of Neurology, School of Medicine, Shiraz University of Medical Sciences, Shiraz, Iran.*

**Keywords:** NR2B-containing NMDAR, Experimental autoimmune encephalomyelitis, RO25-6981

## Abstract

Neurodegeneration is the pathophysiological basis for permanent neurological disabilities in multiple sclerosis (MS); thus neuroprotection is emerging as a therapeutic approach in MS research. Modulation of excitotoxicity by inhibition of NMDARs has been suggested for neuroprotection, but selective antagonisation of the NR2B subtype of these receptors, a subtype believed to play a more pivotal role in neurodegeneration, has not been tested in MS. In this study inhibition of NR2B-containing NMDAR was evaluated on the animal model of MS, experimental autoimmune encephalomyelitis (EAE). EAE induction was done using MOG in C57BL/6 mice. Therapeutic administration of different doses of highly selective NR2B-containing NMDAR inhibitor (RO25-6981) was compared with memantine (non-selective NMDAR antagonist) and vehicle. Neurological deficits in EAE animals were more efficiently decreased by selective inhibition of NR2B-containing NMDARs. Histological studies of the spinal cords also showed decreased inflammation, myelin degradation and neuro-axonal degeneration when RO25-6981was administered with higher doses. The effects were dose dependent. Regarding the role of NR2B-containing NMDARs in excitotoxicity, selective inhibition of these receptor subtypes seems to modulate the neurological disabilities and pathological changes in EAE. Further elucidation of the exact mechanism of action as well as more experimental studies can suggest NR2B-containing NMDAR inhibition as a potentially effective treatment strategy for slowing down the clinical deterioration of disability in MS.

## Introduction

Multiple sclerosis (MS) is a neurodegenerative disease caused by autoimmune responses against myelin in the central nervous system (CNS) (1, 2). Most MS patients experience a relapsing-remitting clinical course; however, some neurological deficits may remain during remissions (3). The pathophysiologic basis for these irreversible neurological deficits is neurodegeneration (4). In progressive forms, neurodegeneration is also a major cause of progressive disabilities in MS patients (5). Neurodegeneration includes axonal loss and cell death. Current MS therapies modulate the immune responses and decrease relapse rate in relapsing remitting MS (RRMS). However, in the long-term, they cannot halt the progression of disability (6). Furthermore, they show limited efficacy in progressive forms of MS (5). Hence, neuroprotective medications are clinically necessary to slow down or prevent the progression of disabilities in all forms of MS. Some neuroprotective compounds have been evaluated in MS animal model, experimental autoimmune encephalomyelitis (EAE) (4). One group of these compounds targets mediators of glutamate excitotoxicity (cell toxicity following excitation conferred by elevated levels of excitatory neurotransmitters like glutamate). Glutamate excitotoxicity is a pathophysiological process believed to play different roles in MS pathogenesis (7). For example, myelin degradation, blood-brain-barrier (BBB) disruption, neurovascular injury, cell death and axonal degeneration are partially attributed to excitotoxicity (8-10). At the cellular level, excitotoxicity results in influx of calcium and subsequent activation of enzymes and signals causing demyelination, cell death and axonal loss (11, 12). It is believed that calcium toxicity is maximal when N-methyl-D-aspartate receptors (NMDARs) are over-stimulated by glutamate during excitotoxicity (11, 13, 14). To modulate excitotoxicity (15), antagonists of NMDARs (Memantine and MK-801) have been tested in EAE. These non-selective NMDAR antagonists were effective in modulation of the disease (16, 17, 18). 

Major subtypes of NMDAR include NR2A and NR2B-containing NMDARs. These subtypes play different roles when they are over-stimulated with glutamate. Excitotoxicity-dependent cell death is a consequence of over-stimulation of NR2B- (but not NR2A-) containing NMDARs (19, 20). Conversely, stimulation of NR2A-containing NMDARs might transfer a pro-survival signal (21). Therefore, the anti-survival effects of excitotoxicity can be modulated by selective inhibition of NR2B-containing NMDARs and simultaneously, the cell continues to be supported by the pro-survival signal transferred by NR2A-containing NMDARs (22). So, although non-selective antagonists of NMDARs have been effective in EAE, selective inhibition of NR2B-containing NMDARs might offer more effective neuroprotection. In this study, the therapeutic effect of selective antagonist of the NR2B-containing NMDARs, Ro 25-6981 (23), has been evaluated in EAE.

## Experimental

*Drugs and biochemicals*

Memantine and myelin oligodendrocyte glycoprotein (MOG) 35-55 were purchased from Alexis Biochemicals (San Diego, CA), complete Freund’s adjuvant (CFA) from Sigma Aldrich (St. Louis, MO) pertussis toxin from ENZO Life Sciences (Farmingdale, NY) and RO 25-6981 ((αR,βS)-α-(4-hydroxyphenyl)-β-methyl-4-(phenylmethyl) were purchased from Tocris Biosciences (Ellisville, MO).

*Animals*

Female C57BL/6 mice (purchased from Pasteur institute, Tehran, Iran) were housed in a pathogen-free room in the laboratory animal center of Shiraz University of Medical Sciences. The animals were kept at 12 h light and 12 hours darkness, temperature of 22 °C and humidity of 30%. All procedures were approved by the Ethics Committee of Shiraz University of Medical Sciences, adhered to the Guide for the Care and Use of Laboratory Animals published through the National Academy Press (Washington D.C. 1996).

*Induction of EAE with MOG*_35–55_

To induce EAE, female mice (9-12 weeks old and 18-22 g weight) were inoculated with 250 μg MOG emulsified in CFA (24). MOG was dissolved in 100 μL phosphate buffered saline (PBS) mixed with 100 μL of CFA supplied with 4 mg/mL heat-killed Mycobacterium tuberculosis. The mixture was subcutaneously injected in the flank of mice under light ether narcosis. Subsequently, 500 ng pertussis toxin was dissolved in 200 μL PBS and administered *intraperitoneally* (*i.p*.) immediately and 48 hours later. One group of mice was inoculated with CFA and PBS mixture without MOG on the day of induction and was labeled as sham group (group 6). 

*Therapeutic administration of experimental medications*

Memantine and RO 25-6981 were dissolved in sterile PBS before injection. Memantine, RO 25-6981 and PBS were administered *i.p*. once daily, after the onset of clinical signs (on day 12) till day 15 post-immunization (*p.i*.). Based on the medication administered, the EAE mice were separated into five groups. Sterile PBS and memantine 20 mg/Kg were administered to the mice in groups 1 and 2 as negative and positive controls, respectively (n=10 in each group). Three doses (3, 10 and 25 mg/Kg/day) of RO 25-6981 were injected to the mice in groups 3 (n=9), 4 (n=10) and 5 (n=9). The 6^th^ group (sham group) received PBS (n=10). 

*Assessment of neurological* *disease severity* *and progression*

Neurological disease severity was graded by daily assessment of neurological score, using standard grading system (25): 0: Normal (No clinical signs). 1: Tail without natural stretch. 2: Complete tail paralysis. 3: Partial hind limbs paralysis. 4: Complete hind limbs paralysis. 5: Four limb paralysis. 6: Death. Mice weights were recorded every 3 days before the onset of the disease and daily after the disease strted. 

*Histopathological evaluation*

On the 15^th^ day post-immunization (*p.i*.), animals were sacrificed and spinal cords were carefully removed and immersed in formalin 10%. Fixed tissues were embedded in paraffin wax and sliced into 5-μm sections. Sections were placed on glass slides and stained with Hematoxylin and Eosin (H and E), Luxol fast blue (LFB) and Bielschowsky's silver impregnation methods for evidence of inflammation, demyelination and axonal degeneration, respectively (24, 26-27). In H and E study, perivascular cuffing and parenchyma infiltration of inflammatory cells were examined. Demyelination was evaluated by LFB which stains the myelin blue. Distortion and dissection of axons as a representative of axonal loss and neuronal degeneration was examined using Bielschowsky's silver impregnation staining (26). Inflammation, demyelination and degeneration were scored using semi-quantitative systems (27-29).

*Statistical analysis*

One way ANOVA and Sheffe post hock tests were used for statistical analysis of means of weight on day 15. Repeated measurement ANOVA was used for evaluation of time-dependent changes in weight. p-value less than 0.05 were accepted as significant. For determination of differences in means of neurological deficits and pathological scores among groups, Kruskal-Wallis test and between two groups, Mann-Whitney U*-*test was used. Bonferroni's correction was applied to adjust the level of significance for non-parametric data (30, 31). Changes in means of neurological scores during the disease course were analyzed using non-parametric Friedman and Wilcoxon tests. SPSS 11.5 software was used for statistical analyses. 

## Results

*Changes in mice body weight during the study*

**Figure 1:**
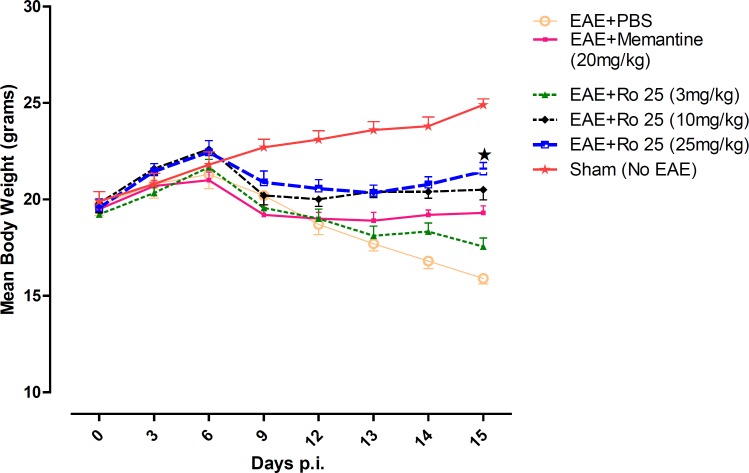
The effect of different doses of RO25-6981 compared to PBS and memantine on changes in mean body weight over time during EAE. All groups (except sham group) showed similar progression of weight loss till day 12, but groups treated with RO 25-6981, specially group 5 (25 mg/Kg/day) displayed gradual recovery of weight from day 12 *p.i*. EAE+Ro 25= EAE groups treated with RO 25-6981 ( Groups 3,4 and 5), EAE+ Memantine= EAE group treated with Memantine (group 2). Each graph presents daily mean body weight ± S.E.M. = Significant compared to PBS

On the first day of the experiment, there was no significant difference among the average weights of the mice in groups. Weight loss was the first sign of disease started in EAE-induced mice. The mice in the sham group (group 6) displayed progressive weight gain. The EAE mice receiving PBS (group 1) lost weight dramatically. RO 25-6981 modified the course of weight loss time-dependently. Modification of weight decrease was more effective with administration of 25 mg/Kg/day of Ro 25-6981 (p < 0.05) during the course of treatment (Figure 1).

*Progression of* *neurological* *disease*

**Figure 2:**
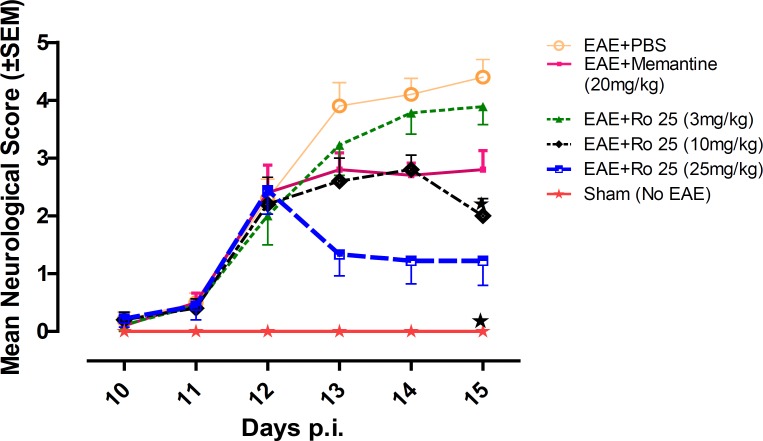
The effect of different doses of RO25-6981 compared to PBS and memantine on changes in daily mean neurological scores over time during EAE. Low dose of Ro 25-6981 (3 mg/Kg/day; group 3) showed no improvement in the course of EAE, but moderate dose (10 mg/Kg/day; group 4) displayed a modest improvement compared to PBS (group 1). Subsequent increase in dose to 25 mg/Kg/day (group 5) demonstrated superior efficacy of RO 25-6981 compared to memantine. EAE+Ro 25= EAE groups treated with RO 25-6981 (Groups 3, 4 and 5), EAE+ Memantine= EAE group treated with Memantine (group 2). Each graph presents daily mean neurological score± S.E.M.  = Significant compared to PBS (Phosphate Buffer Saline) and memantine. EAE= Experimental Autoimmune Encephalomyelitis, SEM=standard error of means, Ro= RO 25-6981.

Mice in group 1 showed a progressive aggravation of neurological disability. Administration of memantine (group 2) resulted in modulation of the disease course compared to PBS (group 1). The lowest dose of RO 25-6981 (group 3) showed no effect in modulation of the disease course; however, two higher doses of RO 25-6981 (groups 4 and 5) resulted in a time-dependent improvement in neurological score compared with PBS (group 1). High dose RO 25-6981 (25 mg/Kg/day; group 5) was superior to memantine (p < 0.001). The effect of RO 25-6981 was both time and dose dependent (Figure 2).

*Neurological disease severity on day 15 p.i*

Early neurological deficits appeared around days 11-12 *p.i*. On day 12 *p.i* (before administration of the experimental drugs) no significant difference existed in mean neurological scores among EAE-induced groups (groups 1 to 5). On the last day of experiment (Day 15) mean neurological scores were significantly different among groups (p = 0.001). The lowest score among EAE-induced groups was recorded in the group treated with 25 mg/Kg/day of RO 25-6981 (group 5), which was significantly less than the controls (groups 1 and 2) as well as the group treated with 3 mg/Kg/day of RO 25-6981 (group 3). When compared with moderate dose of RO 25-6981 (10 mg/Kg/day; group 4), although the score for group 5 was less, considering the Bonferroni's correction, the difference was not considered significant. 

*Histopathological evaluation*

**Figure 3:**
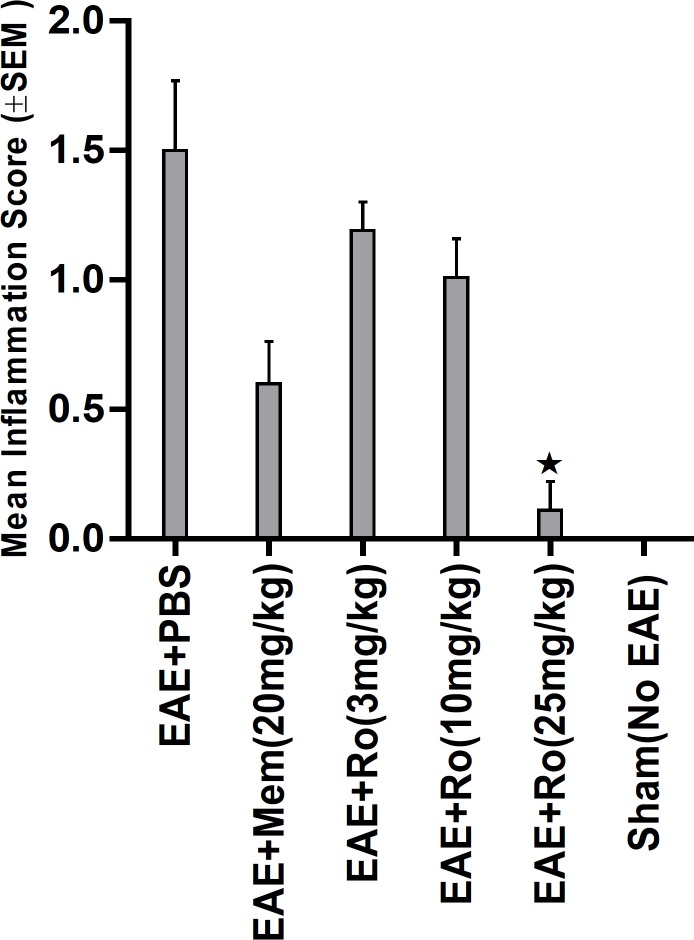
Attributed mean inflammation score assessed by H and E staining of spinal cord sections**.** Therapeutic administration of RO 25-6981 caused a decrease in inflammation in a dose-dependent manner. The highest dose of RO 25-6981 (25 mg/Kg; group 5) displayed a dramatically low score of inflammation.  = Significant compared to PBS and memantine. SEM=standard error of means, Ro= RO 25-6981, Mem=memantine.

By H and E staining, inflammatory score in spinal cord was calculated in each group (Figure 6). There was significant difference among experimental groups regarding this score (p = 0.001). A dramatically low score of inflammation was recorded in the group treated with the highest dose of RO 25-6981 (25 mg/Kg; group 5). Mean inflammation score in this group was not statistically different with the sham group (group 6). Groups receiving moderate and low doses of RO 25-6981 (10 mg/Kg; group 4 and 3 mg/Kg; group 3) also displayed decreased inflammation. The highest dose of RO 25-6981 (25 mg/Kg; group 5) was more efficient in resolution of inflammation compared to memantine (p = 0.006). The mean score of inflammation in all groups is represented in Figure 3.

**Figure 4:**
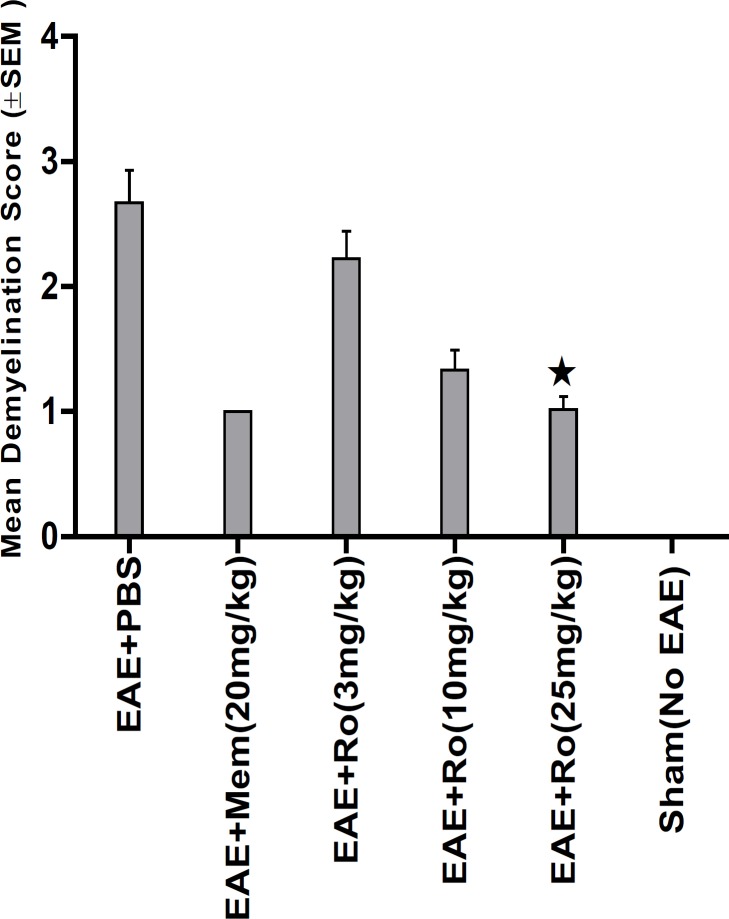
Attributed mean Demyelination score assessed by LFB staining of sections from spinal cord. Demyelination showed dose-dependent decrease with administration of RO 25-6981. High dose of RO 25-6981 decreased demyelination more effectively than low dose.  =Significant compared to PBS. SEM=standard error of means, Ro= RO 25-6981, Mem=memantine

LFB staining (Figure 6) showed lower demyelination score in groups treated with RO 25-6981 in a dose dependent fashion. High dose of RO 25-6981 (25 mg/Kg; group 5) decreased demyelination significantly more than low dose (p = 0.001). However, the difference of demyelination in high-dose group (25 mg/Kg; group 5) with those treated with moderate-dose RO 25-6981 (10 mg/Kg; group 4) or memantine (group 2) was not significant. The mean score of demyelination in all groups is represented in Figure 4. 

**Figure 5:**
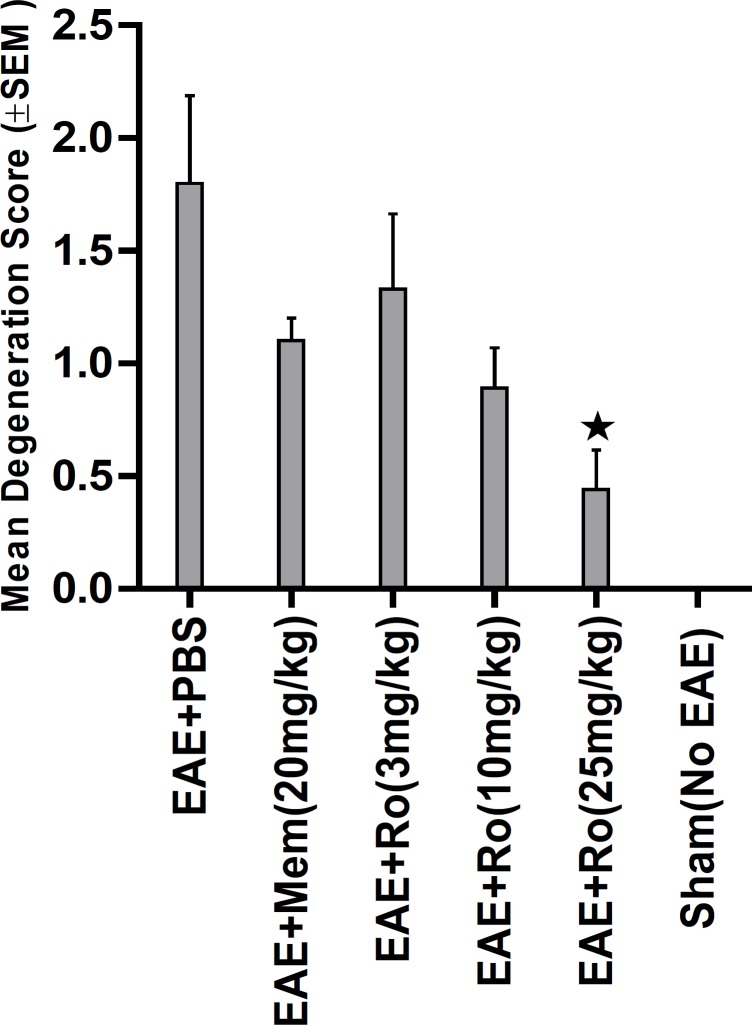
Attributed mean Degeneration score in groups assessed by Bielschowsky staining of sections from spinal cord. Reduction of axonal density and distortion of axons in EAE mice are representative of axonal loss and neuronal degeneration. Degeneration showed dose- dependent decrease with administration of RO 25-6981. High dose of RO 25-6981 decreased degeneration more effectively than low dose.  = Significant compared to PBS and memantine. SEM= standard error of means, Ro= RO 25-6981, Mem=memantine

Bielschowsky's staining (Figure 7) revealed significantly less axonal degeneration in the RO 25-6981-treated groups in a dose dependent manner. High dose of RO 25-6981 (25 mg/Kg; group 5) decreased degeneration more effectively than low dose (3 mg/Kg; group 3). Moderate dose of RO 25-6981 (10 mg/Kg; group 4) also effectively modulated axonal degeneration. The degeneration was significantly lower in high-dose RO 25-6981- treated group (25 mg/Kg; group 5) when compared with group 2 treated with memantine (p=0.008). The mean score of degeneration in all groups is represented in Figure 5.

**Figure 6:**
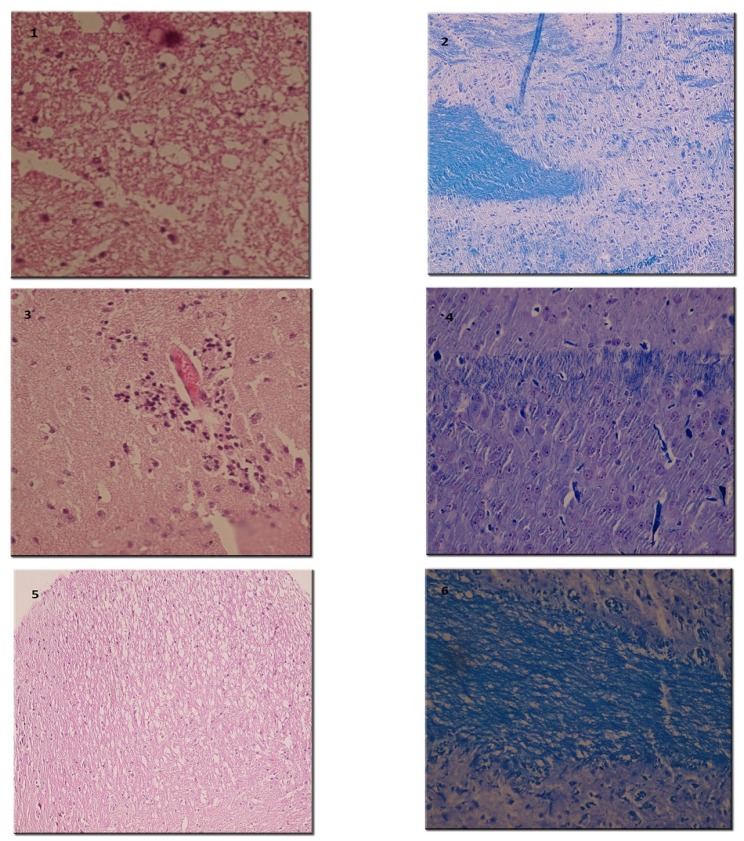
Histopathological evaluation of spinal cords by H&E and LFB. (1) Vacuolation and myelin degradation (H&E x 250). (2) Myelin degradation (LFB x 250). (3) Perivascular lymphocytic infiltration (H&E x 250). (4) Active myelin degradation with infiltration of macrophages. (H and E x 250). (5) Low power view of high-dose Ro 25-6981-treated mouse spinal cords with resolution of inflammation with no macrophage (H&E x 100). (6) Relatively normal myelin fibers after treatment with high-dose Ro 25-6981 (LFB x 250).

**Figure 7:**
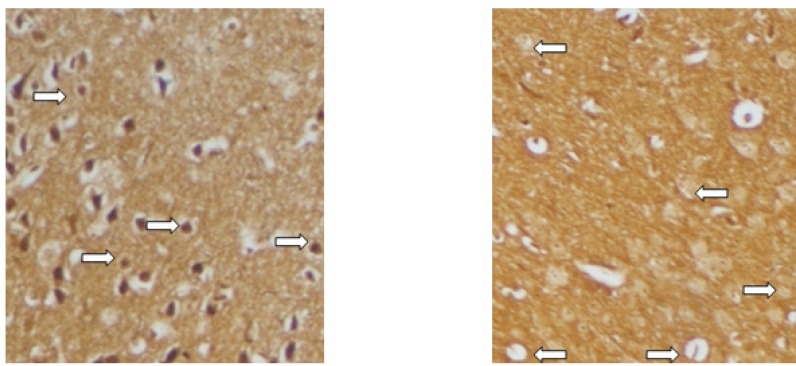
Histopathological evaluation of spinal cord by Bielschowsky's stain. Relatively normal axons (left) after treatment with high-dose Ro 25-6981 and axonal loss (right) in non-treated EAE mice

## Discussion

Although neurodegeneration in MS was described by Charcot, the pioneering introducer of the disease in 1868, there is no approved medication mechanistically designed on the basis of this pathological event. In recent decades, monitoring of disability progression in patients treated with approved medications has shown the need for better medications which can protect the cells and axons in the CNS (32). Therefore, finding neuroprotective agents to slow down or prevent disability progression has become a priority in MS research. Novel agents targeting the neurodegenerative process in MS are being examined in MS models (32). One group of these neuroprotective agents interrupt the pathophysiolical processes involved in cell death and axonal loss (4). Excitotoxicity has been associated with axonal loss and cell death in many acute neurological insults and chronic neurodegenerative states including MS (4, 8-10). The effect of excitotoxicity initiates from the first days of the disease course in MS (10). Glutamate is considerably elevated in the CNS. Elevated concentration of glutamate leads to activation of different ionotropic receptors opens their ion channels and significantly increases the influx of calcium ions into the cells. High concentration of intracellular calcium induces activation of many enzymes and signaling cascades which might cause neuronal cell death and axonal loss (11, 12). It has been proposed that neuronal injury is more efficiently triggered when calcium ions enter neurons at specific entry points (11, 13, 14). According to this hypothesis, NMDARs are major routs of calcium influx in glutamate excitotoxicity causing prolonged calcium build-up in the neural cells and cell death. NMDAR consists of an NR1 subunit combined with a variety of NR2A to NR2D and NR3 subunits. During the course of EAE, NR2A and NR2B subunits have been reported to up-regulate in CNS tissues (33, 34). Cell death might be a consequence of activation of NR2B-containing NMDARs by glutamate during excitotoxicity (19). In contrast to NR2B, activation of NR2A-containing NMDARs can protect the cells by transferring pro-survival signals (21). Calcium-dependent cell injury can be modulated by selective inhibition of NR2B-containing NMDARs without inhibition of NR2A-containing NMDARs which probably protects the cell during excitotoxicity. Neuroprotection by some medications has been attributed to inhibition of NR2B-containing NMDARs (35). To find more effective neuroprotective agents with fewer adverse effects, NR2B-containing NMDARs antagonists have been tested in some diseases (21, 36). In MS, however, only non-selective NMDAR antagonists have been evaluated. 

In this study the effect of inhibition of the NR2B-containing NMDARs was evaluated in EAE using a highly selective and potent antagonist of this receptor subtype, RO 25-6981 (23). RO 25*-*6981 is a potent and selective blocker of NMDARs containing the NR2B subunit. The selectivity of Ro 25-6981 for inhibition of NR2B-containing NMDARs is 5000-fold more than inhibition of NR2A-containing NMDARs. In the first step of a preclinical drug screening study, several doses of this antagonist were compared with the vehicle (PBS) as negative control and memantine, a non-selective antagonist of NMDAR as positive control. As menantine shows therapeutic effects in acute EAE by inhibition of NMDARs, we used it as a control drug in this study (37). Memantine is a clinically prescribed NMDAR antagonist under clinical trials on several neurodegenerative diseases or acute CNS catastrophes; however, the search for new and more potent NMDAR modulators with greater efficacy is ongoing (38). In animal models of MS memantine has been effective in abrogation of neurological deficits and modulation of the disease course when administered either prophylactically (concurrent with induction of EAE) or therapeutically (just after the onset of the disease) (17, 18, 37). Menantine has been studied in clinical phases II and III of several clinical trials related to MS. Currently, memantine is under further MS trials (39-42). 

In our study, the therapeutic administration of drugs was started when initial signs were seen in EAE-induced mice. A non-induced group was also monitored continously to compare the changes with induced groups. 

 In our study, it was found that pharmacological inhibition of NR2B-containing NMDARs may have beneficial effects in modulation of neurological deficits. In terms of suppression of clinical disease progression, the therapeutic effect of RO 25-6981 when administered with the highest dose was more than all other experimental drugs including memantine. Moderate dose of RO 25-6981 was also effective in disease suppression. The extent of disease suppression by this dose was less than the high dose, although considering the Bonferroni's correction, the difference was not significant. The weight was also less decreased with administration of RO 25-6981*. *EAE mice start progressive weight loss from day 6 *p.i*. All groups showed a similar trend of weight loss till day 12, but groups under treatment with RO 25-6981, especially group 5 (treated with 25 mg/Kg/day) displayed gradual recovery of weight from day 12 *p.i*. This effect was dose dependent. On day 15, the mean weight in groups was significantly different. High-dose RO 25-6981 was more effective in this regard. 

Pathological study of spinal cords from distinct groups revealed different extents of inflammation, myeline degradation and axoinal loss. Inflammation was significantly lower in the spinal cords of the RO 25-6981-treated EAE-mice. High-dose RO 25-6981 effectively resolved infiltration of inflammatory cells in the spinal cords of the EAE mice. Memantine was less effective in decreasing inflammation. This suggests superior efficacy of NR2B inhibition compared to non-specific antagonisation of NMDARs. RO 25-6981 was dose dependently effective in decreasing myelin degeneration. High dose of RO 25-6981 decreased demyelination significantly more than low dose. Compared with memantine, however, this therapeutic effect was not significantly different. 

Sevierity of axonal degeneration was less in the groups treated with RO 25-6981. This highly selective antagonist shows efficacy in decreasing neuroaxonal injury or prevention of axonal loss. This effect can be mechanistically attributed to modulation of excitotoxicity. NR2B-containing NMDARs are believed to play a pivotal role in excitotoxicity (43). The therapeutic effect of RO 25-6981 can be secondary to modulation of NR2B-containing NMDARs. The stimulatory effect of RO 25-6981 on neurogenesis could also be a reason (44).

Moreover, the effect of RO 25-6981 could be partially attributable to decrease of inflammation in the CNS. This inflammation-decreasing effect could be a consequence of modulation of BBB disruption by NR2B-containing NMDAR inhibition. Excitotoxicity has been proposed to contribute to the pathogenesis of BBB break-down via NMDARs in endothelial cells (7, 45). Our study supports this idea, because modulation of EAE course and axonal loss by the specific antagonist of NR2B subtype is simultaneous with dramatic resolution of inflammatory cell infiltration in the spinal cord. There are no published reports studying the effect of NR2B-containing NMDARs on BBB disruption in EAE, and the validity of this probable mechanism needs to be investigated independently. 

Compared to memantine, RO 25-6981 appears to be more protective against EAE disease progression and histopathological evidence regarding inflammation and axonal degeneration. The superior efficacy of Ro 25-6981 is attributable to the pharmacological characteristics of this selective antagonist: much higher efficiency of RO 25-6981 in blocking NR2B-containing NMDARs, lack of inhibitory effect on NR2A-containing NMDARs, and its higher potency compared to memantine (23, 46). 

It is reported here that short-term treatment of EAE with antagonists of NR2B subtype could be effective in terms of disease modulation and pathological changes. Long-term effects remain to be studied. The biological biomarkers of axonal loss and cell death can be followed in other studies for further evaluation of the mechanism of action. Preventive administration of Ro 25-6981 can also help in understanding the role of NR2B-containing NMDARs in EAE development, as a prerequisite to proposing hypotheses about human MS. Our findings could be used as a cornerstone for hypothesizing theories and designing complementary studies to validate NR2B-containing NMDARs as possible targets in pharmacotherapy of MS. 
